# Characterization of polysaccharide from *Lonicera japonica* Thunb leaves and its application in nano-emulsion

**DOI:** 10.3389/fnut.2023.1248611

**Published:** 2023-08-09

**Authors:** Yongchao Li, Benguo Liu, Jing Yang, Junliang Sun, Junjian Ran, Xinhong Liang, Yinglin Li

**Affiliations:** ^1^School of Life Sciences, Henan Institute of Science and Technology, Xinxiang, China; ^2^Henan International Joint Laboratory of Plant Genetic Improvement and Soil Remediation, Xinxiang, China; ^3^School of Food Science, Henan Institute of Science and Technology, Xinxiang, China

**Keywords:** honeysuckle leaves, nano-emulsion, ultrasonic treatment, process optimization, β-carotene

## Abstract

The polysaccharides in honeysuckle leaves (PHL) were separated and characterized for the first time. The nano-emulsion stabilized by PHL and whey protein isolate (WPI) were also fabricated based on the ultrasonic method. The results indicated that PHL was mainly composed of glucose (47.40 mol%), galactose (19.21 mol%) and arabinose (20.21 mol%) with the weight-average molecular weight of 137.97 ± 4.31 kDa. The emulsifier concentration, WPI-to-PHL ratio, ultrasound power and ultrasound time had significant influence on the droplet size of PHL-WPI nano-emulsion. The optimal preparation conditions were determined as following: emulsifier concentration, 1.7%; WPI/PHL ratio, 3:1; ultrasonic power, 700 W; ultrasonic time, 7 min. Under the above conditions, the median diameter of the obtained nano-emulsion was 317.70 ± 5.26 nm, close to the predicted value of 320.20 nm. The protective effect of PHL-WPI emulsion on β-carotene against UV irradiation was superior to that of WPI emulsion. Our results can provide reference for the development of honeysuckle leaves.

## 1. Introduction

Honeysuckle (*Lonicera japonica* Thunb) is an important medicinal and edible plant. Its flowers are rich in chlorogenic acid, luteolin, and polysaccharide, which are widely used in traditional Chinese medicine to treat cold, respiratory tract infection, sore throat and headache (1–3). The food industry also often uses honeysuckle flowers to produce plant drinks or tea alternatives (4). During the process of honeysuckle growth, in order to improve the tree shape and increase the yield of flowers, it is necessary to trim its branches and leaves 3–4 times a year, resulting in a large number of discarded fresh leaves. Although its yield is about 10 times that of flowers, it has been regarded as waste material. However, studies have shown that the bioactive components and pharmacological effects of honeysuckle leaves are similar to those of flowers, and they are also rich in polyphenols such as chlorogenic acid and luteolin (5, 6). However, the related research on polysaccharides from honeysuckle leaves (PHL) is rarely reported.

The common bioactive components in food have poor stability, aqueous solubility and bioavailability, and are easily affected by environmental factors and gastrointestinal digestion (7, 8). Therefore, it is very important to develop an edible delivery system for these valuable bioactive components. Nano-emulsion is an effective method for encapsulating, protecting and promoting the absorption of bioactive components (9). Small molecular surfactants, such as Tween and Span, are widely used in nano-emulsions because of their ability to expand easily at the oil–water interface and reduce surface tension. However, the use of these chemically synthetic surfactants has aroused consumers’ concern about food safety (10, 11). Therefore, researchers began to explore the use of natural biological macromolecules as emulsifiers in the preparation of nano-emulsions, such as β-lactoglobulin, whey protein isolate, starch, xanthan gum. (12–14). Natural polysaccharides, such as Arabic gum and gum ghatti, have certain emulsifying activity. They are usually less affected by environmental factors, but the droplets of emulsions that they stabilize are larger. Therefore, it is necessary to cooperate with proteins to further improve their emulsifying performance (15, 16).

The preparation method of nano-emulsion of food mainly adopts the method of high-energy emulsification, which relies on the equipment to produce destructive forces such as high-speed shear and impact, break the interface between oil and water, and make large droplets disperse, break and fine in a short time (17). With the increase of the interface area, the emulsifier is absorbed to the newly formed interface in time to stabilize the emulsion. In general, the primary emulsion is prepared, and then the nano-emulsion is obtained by further emulsification and homogenization (18). Common high-energy emulsifying methods include high-pressure homogenization, dynamic high-pressure microfluidization, and ultrasonic emulsification. Ultrasonic emulsification is a new technology, which is widely used to improve the properties and quality of food (19). When it is used in the preparation of nano-emulsion, ultrasonic produces strong mechanical vibration and cavitation to break the oil–water phase interface. The prepared nano-emulsion has small particle size, narrow particle size distribution and high stability. This method has the advantages of simple process, low amount of surfactant, low production cost and no environmental pollution (20, 21).

In summary, this study intended to separate and characterize polysaccharides in honeysuckle leaves (PHL) for the first time. The nano-emulsion was prepared by ultrasonic treatment with PHL and whey protein isolate (WPI) as emulsifier, and the preparation process was optimized. The results obtained can promote the development and utilization of honeysuckle leaves and provide reference for the construction of new food-grade nano-emulsion system.

## 2. Materials and methods

### 2.1. Materials and chemicals

The leaves of honeysuckle (variety, Baijin 2) were collected from the experimental field of Henan Institute of Science and Technology (Xinxiang, China) in October 2022. Fucose (Fuc), rhamnose (Rha), arabinose (Ara), galactose (Gal), glucose (Glc), xylose (Xyl), mannose (Man), fructose (Fru), ribose (Rib), galacturonic acid (Gal-UA), glucuronic acid (Glc-UA), manuronic acid (Man-UA), guluronic acid (Gul-UA) were purchased from Sigma-Aldrich (St. Louis, MO, United States). The other chemicals were of analytical grade.

### 2.2. Extraction of PHL

The naturally air-dried honeysuckle leaves was crushed with a high-speed multifunctional grinder (Kemanshi 304, Yongkang, China) and passed the 80 meshes sieve. According to a published method (22), the sample powder (40 g) were mixed with 2000 mL ethanol at 50°C for 2 h, then centrifuged. The decolorized powder was further extracted with 800 mL distilled water at 100°C for 0.5 h, and then centrifuged. Four times the volume of ethanol was added to the supernatant and left for 24 h. The precipitate was dissolved again by boiling water, and after cooling, 4 times the volume of ethanol was added and left standing for 24 h. The precipitate was collected and vacuum-dried. Finally, 2.01 g PHL was obtained.

### 2.3. Determination of molecular weight of PHL

According to a previous report (23), the molecular weight of PHL was determined by a size exclusion chromatography - multi angle laser light scattering - refractive index system (SEC-MALLS-RI), including a U3000 HPLC (Thermo, United States), a DAWN HELEOS II MALLS detector (Wyatt technology, USA) and an Optilab T-Rex RI detector (Wyatt technology, USA). The concentration of PHL solution was 1.0 mg/mL and the injection volume was 100 μL. The mobile phase was 0.1 M NaNO_3_ aqueous solution, and the flow rate was 0.5 mL/min. The separation of the sample was carried out at 45°C on a series of SEC columns: Ohpak SB-805 HQ (300 × 8 mm), Ohpak SB-804 HQ (300 × 8 mm) and Ohpak SB-803 HQ (300 × 8 mm).

### 2.4. Determination of monosaccharide composition of PHL

5 mg PHL was hydrolyzed with 1 mL 2 mol/L trifluoroacetic acid solution (TFA) at 121°C for 2 h, dried with nitrogen, and redissolved with ultrapure water. The obtained sample solution was analyzed by an ICS5000+ high-performance anion-exchange chromatography (Thermo Fisher Scientific, United States) with a pulsed amperometric detector (24). A CarboPac PA-20 anion-exchange column was used for separation at 30°C. The mobile phase was composed of A (H_2_O), B (0.1 M NaOH), and C (0.1 M NaOH, 0.2 M NaAc). The flow rate was set at 0.5 mL/min. The gradient program was as following: volume ratio of A, B, C was 95:5:0 at 0 min, 85:5:10 at 26 min, 85:5:10 at 42 min, 60:0:40 at 42.1 min, 60:40:0 at 52 min, 95:5:0 at 52.1 min, 95:5:0 at 60 min. The data were record and treated by Chromeleon 7.2 CDS software.

### 2.5. Preparation of PHL-WPI nano-emulsion

The PHL-WPI nano-emulsion was prepared according to a previous report (25). A certain amount of PHL and WPI were dissolved in water according to the designed PHL/WPI mass ratio. The obtained aqueous solution was mixed with corn oil at the oil to water ratio of 1:19. The mixture was homogenized at 15000 r/min for 1 min to obtain PHL-WPI primary emulsion. It was further treated by a SCIENTZ JY99-IIDN ultrasonic homogenizer (Ningbo, China) at a designated power for a specific time to obtain PHL-WPI nano-emulsion.

### 2.6. Measurement of droplet size of nano-emulsion

The nano-emulsion was diluted properly and analyzed by a Nano-ZS laser nanoparticle size analyzer (Malvern Instruments, Worcestershire, United Kingdom) to determine its droplet size distribution (26).

### 2.7. Single factor experiment on the preparation of nano-emulsion

With the median droplet size of the nano-emulsion as the evaluation index, the effects of the WPI-to-PHL mass ratio (1:1, 2:1, 3:1, 4:1, 5:1), the emulsifier (PHL + WP) concentration (0.5, 1.0, 1.5, 2.0, 2.5%), ultrasound power (300 W, 400 W, 500 W, 600 W, 700 W, 800 W) and ultrasonic time (2 min, 4 min, 6 min, 8 min, 10 min) on the preparation of nano-emulsion were investigated.

### 2.8. Optimization of preparation process of nano-emulsion

On the basis of single factor experiment, the response surface methodology (RSM) was used for optimization of the preparation process of nano-emulsion. RSM uses multiple regression equations to fit the function relationship between factors and response values, and seeks the optimal process parameters through the analysis of regression equations (27). In this study, four factors, emulsifier concentration (X_1_), WPI-to-PHL mass ratio (X_2_), ultrasonic power (X_3_), and ultrasonic time (X_4_), were selected as independent variables, and the median particle size (Y) of nano-emulsion was taken as the response value. The experimental design was shown in Table 1.

**Table 1:** Monosaccharide composition of polysaccharides in honeysuckle leaves (PHL).

	Composition (mol%)		Composition (mol%)
Fuc	0.47 ± 0.03	Xyl	2.54 ± 0.20
Rha	3.77 ± 0.25	Man	2.44 ± 0.06
Ara	20.21 ± 0.19	Gal-UA	2.32 ± 0.09
Gal	19.21 ± 0.23	Gul-UA	0.55 ± 0.04
Glc	47.40 ± 0.27	Glc-UA	1.08 ± 0.05

### 2.9. Evaluation of β-carotene protection capacity of nano-emulsion

β-Carotene was dissolved in corn oil (2 mg/mL), and PHL-WPI nano-emulsion containing β-carotene was prepared according to the optimum conditions obtained in section 2.8. The emulsion was placed in an incubator at 30°C and stored at 15 cm under the UV lamp (6 W). The 1 mL emulsion was taken out periodically and mixed with 10 mL of a mixture of ethanol and n-hexane (2:3, v/v). The mixture was vortexized for 1 min and left for 10 min. The absorbance of supernatant at 450 nm was recorded to calculated β-carotene retention rate with β-carotene-loaded corn oil and WPI emulsion as control (28).

### 2.10. Statistical analysis

The results were expressed as mean ± standard deviation (*n* = 3). The statistical comparison was based on Duncan’s test with a confidence level of 95%. SPSS 18 software was used for statistical analysis. Design-Expert software was used to design and analyze the RSM experiment.

## 3. Results and discussion

### 3.1. Structural characterization of PHL

**Figure 1:**
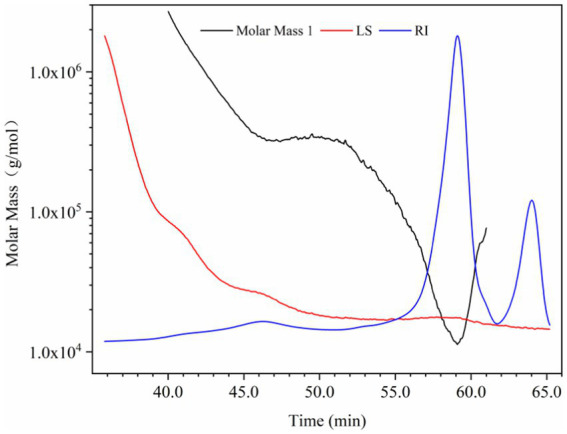
Size exclusion chromatography (SEC) - multi angle laser light scattering (MALLS) - refractive index (RI) analysis of polysaccharides in honeysuckle leaves (PHL).

At present, there is no report on the structural identification of PHL, but there are many studies on polysaccharides of honeysuckle flowers. Zhang et al. separated the water-soluble polysaccharides of honeysuckle flowers into one neutral fraction and four acidic fractions. It was found that the neutral fraction was a starch-like glucan with some arabinogalactan. The acidic components were pectic polysaccharides with the molecular weight of 19–383.8 kDa, which were composed of galacturonic acid, galactose and arabinose (29). Lin isolated a pectin from honeysuckle flowers. Its molecular weight was 54 kD, which was composed of rhamnose, galacturonic acid, galactose and arabinose in the molar ratio of 10.77: 7.88: 15.45: 65.89 (30). Liu et al. purified an active polysaccharide (LFA03-a) from honeysuckle flowers by DEAE-cellulose column and Sephacryl S-300 column, and found that it had a rhamnogalacturonan I (RGI) backbone, which was composed of rhamnose, arabinose, galactose and galacturonic acid with a molar ratio of 18.1: 25.3: 36.8: 19.5 (31). In this study, the SEC-MALLS-RI system was used to determine the molecular weight of PHL. As shown in Figure 1, the red line represents the MALLS signal, whose intensity is proportional to the molecular size and molecular weight of the sample. The blue line indicates the RI signal, which is related to the sample concentration. The black line is the molecular weight fitted based MALLS and RI signals. The weight-average molecular weight (M_w_) and hydration radius (R_w_) of PHL were 137.97 ± 4.31 kDa and 45.10 ± 4.06 nm, respectively. The monosaccharide composition of PHL is shown in Table 1. PHL was mainly composed of glucose (47.40 mol%), galactose (19.21 mol%) and arabinose (20.21 mol%). Compared with the previous reports, we concluded that the composition of polysaccharides of honeysuckle flowers was closer to pectin and significantly different from PHL, which could be caused by the difference in plant tissue structure.

### 3.2. The factors affecting preparation of PHL-WPI nano-emulsion

**Figure 2:**
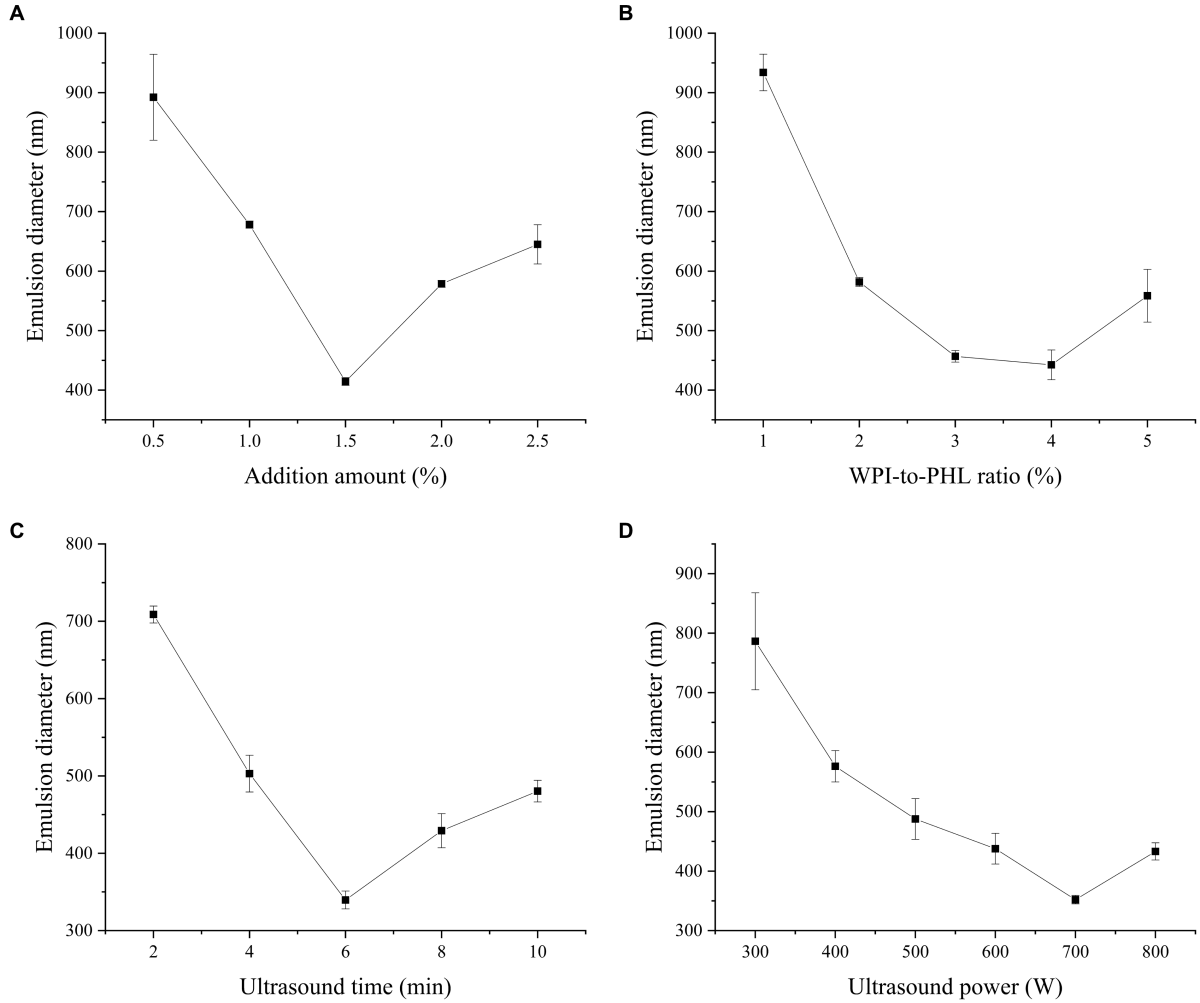
Effect of emulsifier concentration (*c*), WPI-to-PHL ratio (*r*), ultrasound time (*t*) and ultrasound power (*p*) on the median diameter of the emulsion [**(A)** samples developed at *c* = 0.5, 1.0, 1.5, 2.0, 2.5%, *r* = 2, *t* = 2 min, *p* = 600 W; **(B)** samples developed at *r* = 1, 2, 3, 4, 5, *c* = 1.5%, *t* = 2 min, *p* = 600 W; **(C)** samples developed at *t* = 2 min, 4 min, 6 min, 8 min, 10 min, *c* = 1.5%, *r* = 2, *p* = 600 W; **(D)** *p* = 300 W, 400 W, 500 W, 600 W, 700 W, 800 W; *c* = 1.5%, *r* = 2, *t* = 2 min].

WPI has high digestibility and is rich in essential amino acids, which is a natural protein resource with great nutritional value. Because of its amphiphilic nature, WPI can form a viscoelastic film on the oil–water interface to resist various mechanical pressures and keep the emulsion stable (32). It is a high-quality natural emulsifier. Polysaccharide is an important component of plant tissue and has a variety of biological activities, but the emulsifying and stability of polysaccharide-based stable emulsion is poor in practical application. Previous studies have shown that WPI can improve the emulsifying ability of polysaccharide and stabilize emulsion synergistically. Esfanjani et al. fabricated a stable W/O/W emulsion with WPI and pectin to encapsulate the saffron extract (33). Cabezas et al. indicated that the nanoparticles composed of WPI and soybean polysaccharide could construct emulsion systems with outstanding freeze–thaw stability (34). Therefore, the combined use of PHL and WPI provides the possibility for the construction of a safe, healthy and stable emulsion system. During the process of ultrasonic modification, the transient bubble will form rapidly in the vibration period and collapse violently when it reaches the critical size, which enhances the local pressure and produces high shear force. Under this high energy force, the droplet size and interfacial tension of the emulsion can be effectively reduced, and the overall stability of the emulsion can be improved (35). In this study, the nano-emulsion developed by WPI and PHL was prepared base on the ultrasonic emulsification method, and the related influencing factors were investigated. Figure 2A shows the effect of emulsifier concentration (WPI + PHL) on the droplet size of emulsion. With the increase of emulsifier concentration, the average particle size of nano-emulsion decreased. When the concentration was 1.5%, the average droplet size of nano-emulsion was the smallest (414.4 nm), and the further increase of concentration led to the increase of droplet size. This is due to the following reasons: (1) the viscosity of the system increases, which is not conducive to emulsification; (2) at high concentrations, polysaccharides and proteins aggregate, resulting in a decrease in the stability of the emulsion system. The interaction between protein and polysaccharide is sensitive to the ratio of protein and polysaccharide. The change of the ratio will affect the existing state and adsorption of the protein-polysaccharide system, and then change the flocculation rate of the emulsion droplets, resulting in the change of the emulsion properties (36). As shown in Figure 2B, when the WPI/PHL ratio is 4:1, the emulsion droplet size is the smallest (442.47 nm). Figure 2C demonstrates the effect of ultrasonic time on the droplet size of the emulsion. In a certain time range, the droplet size of the emulsion declined gradually with the extension of ultrasonic time, and the droplet size reached the minimum (339.63 nm) at 6 min. This is because the cavitation effect of ultrasound can promote the fragmentation of oil droplets, the uniform dispersion of PHL and WPI, and enhance the oil–water interface area and the interface contact frequency of the emulsified matrix; at this time, PHL and WPI are rapidly adsorbed to the oil–water interface, and the emulsifying property of the system is improved. However, too long ultrasonic time (8 min and 10 min) will reunite the protein and lead to the aggregation of droplets, which is not conducive to the stability of the emulsion. In Figure 2D, the emulsion droplet size declines at first and then ascends with the change of ultrasonic power, reaching the minimum (351.97 nm) at 700 W. Proper ultrasonic power can effectively dissolve the self-aggregation or co-aggregation products of macromolecules such as protein and pectin, thus promoting the interfacial movement. In addition, it is found that the cavitation effect provided by ultrasound can also change the structure of proteins and polysaccharides, promote their electrostatic interaction, and improve the emulsifying properties of the complexes (37). However, the excessive ultrasonic power makes the protein structure highly extended, a large number of hydrophobic groups embedded in the molecules are exposed, and the proteins are easy to aggregate through hydrophobic interaction (38), which leads to the decrease of emulsion stability and the increase of emulsion droplet size.

### 3.3. Optimization of preparation process of PHL-WPI nano-emulsion

In this study, the preparation process of PHL-WPI nano-emulsion was optimized by RSM with ultrasonic time, ultrasonic power, WP/IPHL ratio and emulsifier concentration as independent variables, and the median diameter of nano-emulsion as the response value. The design and results of RSM are shown in Table 2. The results of second order polynomial regression equation (Table 2) and variance analysis (Table 3) suggested that the obtained regression terms were significant. Moreover, the *p* value of “Lack of Fit” (0.6019) was >0.05, indicating that there was a significant correlation between the variables and response value of the fitting equation. The *R*^2^ and Adj *R*^2^ of the fitting model equation were 0.9568 and 0.9064, respectively, which suggested that this equation could be used for experimental prediction. Within the experimental range, all the factors had a significant effect on the median diameter of the emulsion. According to the F test, the influencing effects were as follows: emulsifier concentration > ultrasonic time > WPI/PHL ratio > ultrasonic power.

**Table 2:** RSM Experimental design and results.

Run	X_1_ (%)	X_2_	X_3_ (W)	X_4_ (min)	Y (nm)	Run	X_1_(%)	X_2_	X_3_ (W)	X_4_ (min)	Y (nm)
1	1.5 (0)	3 (0)	700 (0)	6 (0)	343.10 ± 15.82	18	1.5 (0)	2 (−1)	700 (0)	4 (−1)	441.17 ± 19.23
2	1.5 (0)	2 (−1)	700 (0)	8 (1)	342.83 ± 10.47	19	2 (1)	3 (0)	700 (0)	8 (1)	348.40 ± 25.55
3	1.5 (0)	4 (1)	800 (1)	6 (0)	339.83 ± 18.10	20	1.5 (0)	3 (0)	700 (0)	6 (0)	321.43 ± 4.22
4	1.5 (0)	3 (0)	600 (−1)	8 (1)	365.07 ± 5.80	21	1 (−1)	3 (0)	700 (0)	8 (1)	437.93 ± 4.24
5	1 (−1)	3 (0)	700 (0)	4 (−1)	448.93 ± 42.86	22	1.5 (0)	3 (0)	800 (1)	8 (1)	349.90 ± 7.20
6	2 (1)	3 (0)	700 (0)	4 (−1)	395.03 ± 10.59	23	1 (−1)	3 (0)	800 (1)	6 (0)	421.87 ± 62.39
7	1.5 (0)	4 (1)	600 (−1)	6 (0)	377.50 ± 12.05	24	1.5 (0)	2 (−1)	800 (1)	6 (0)	403.70 ± 12.50
8	2 (1)	4 (1)	700 (0)	6 (0)	335.20 ± 11.53	25	2 (1)	2 (−1)	700 (0)	6 (0)	361.70 ± 8.44
9	1 (−1)	2 (−1)	700 (0)	6 (0)	455.20 ± 23.38	26	2 (1)	3 (0)	800 (1)	6 (0)	358.330 ± 3.56
10	1.5 (0)	2 (−1)	600 (−1)	6 (0)	423.20 ± 25.69	27	1 (−1)	4 (1)	700 (0)	6 (0)	397.50 ± 37.11
11	1.5 (0)	4 (1)	700 (0)	4 (−1)	363.60 ± 21.88	**Credibility analysis of the regression equation**					
12	2 (1)	3 (0)	600 (−1)	6 (0)	351.50 ± 16.20	C.V.%	3.4	*R* ^2^	0.9568	Pred *R*^2^	0.7767
13	1 (−1)	3 (0)	600 (−1)	6 (0)	455.20 ± 44.57	PRESS	10533.56	Adj *R*^2^	0.9064	Adeq precision	13.780
14	1.5 (0)	3 (0)	700 (0)	6 (0)	345.00 ± 17.81	**Second-order polynomial equation**					
15	1.5 (0)	4 (1)	700 (0)	8 (1)	336.50 ± 19.13	Y = 336.51–38.87×_1_–23.14×_2_–10.38×_3_–24.68×_4_ + 7.80X_1_X_2_ + 10.04X_1_X_3_–8.91X_1_X_4_–4.54X_2_X_3_ + 17.81X_2_X_4_ + 2.62X_3_X_4_ + 38.50×_1_^2^ + 14.90×_2_^2^ + 26.93×_3_^2^ + 24.83×_4_^2^					
16	1.5 (0)	3 (0)	600 (−1)	4 (−1)	426.87 ± 26.09						
17	1.5 (0)	3 (0)	800 (1)	4 (−1)	401.20 ± 11.21						

X_1_, emulsifier concentration; X_2_, WPI-to-PHL ratio; X_3_, ultrasound power; X4, ultrasound time; Y, median diameter; RSM, response surface methodology.

**Table 3:** ANOVA results.

Source	Sum of squares	df	Mean square	*F* value	*p* value
Model	45125.98	14	3223.28	18.98	<0.0001
X_1_	18132.60	1	18132.6	106.79	<0.0001
X_2_	6424.9	1	6424.9	37.84	<0.0001
X_3_	1291.69	1	1291.69	7.61	0.0173
X_4_	7309.56	1	7309.56	43.05	<0.0001
X_1_X_2_	243.36	1	243.36	1.43	0.2544
X_1_X_3_	403.34	1	403.34	2.38	0.1492
X_1_X_4_	317.43	1	317.43	1.87	0.1966
X_2_X_3_	82.51	1	82.51	0.49	0.499
X_2_X_4_	1268.55	1	1268.55	7.47	0.0182
X_3_X_4_	27.56	1	27.56	0.16	0.6941
X_1_^2^	7907.04	1	7907.04	46.57	<0.0001
X_2_^2^	1183.39	1	1183.39	6.97	0.0216
X_3_^2^	3866.43	1	3866.43	22.77	0.0005
X_4_^2^	3289.04	1	3289.04	19.37	0.0009
Residual	2037.65	12	169.8		
Lack of fit	1694.83	10	169.48	0.99	0.6019
Pure error	342.81	2	171.41		
Cor total	47163.62	26			

ANOVA, analysis of variance.

**Figure 3:**
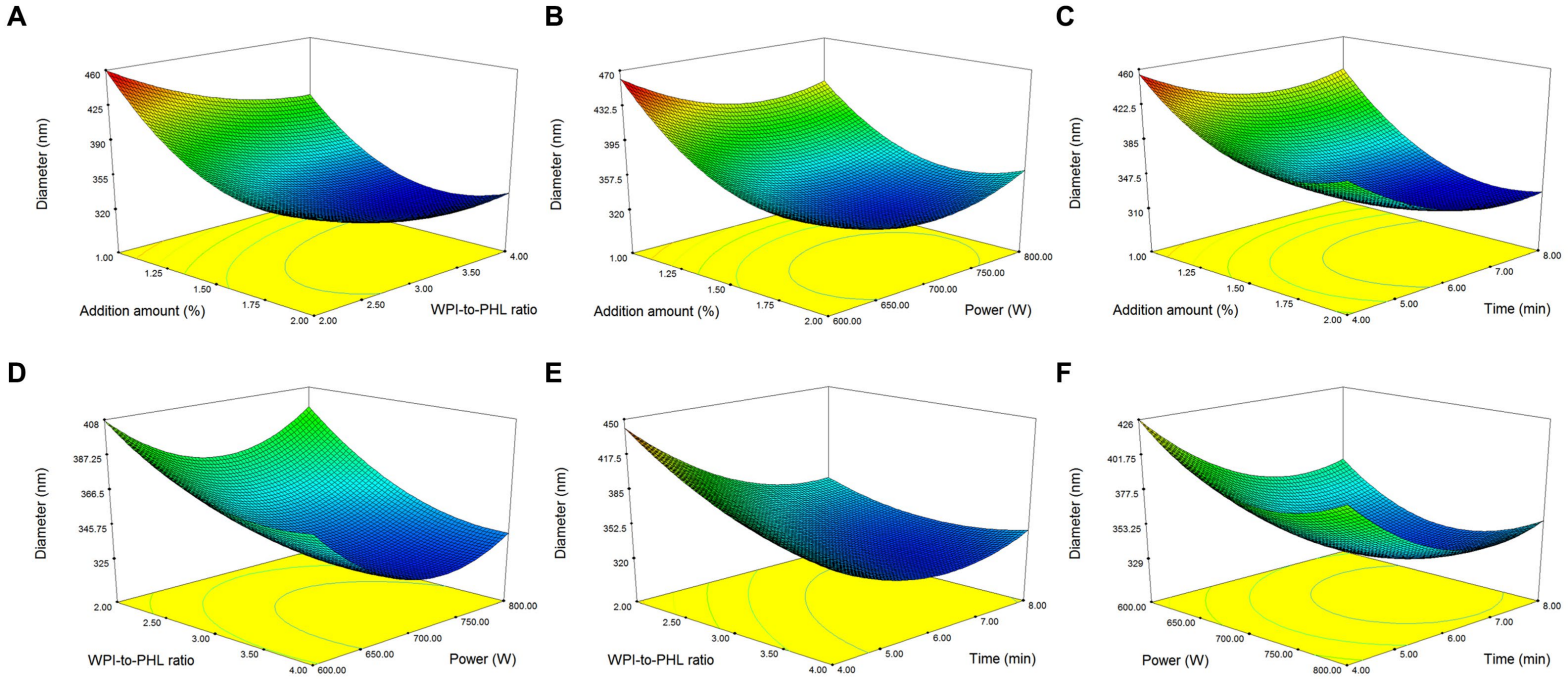
Response surface plots for the ultrasonic preparation of PHL-WPI nano-emulsion.

The mutual influence of factors on the response value is shown in Figure 3. In Figures 3A–C, the increase of emulsifier concentration was beneficial to the rapid decrease of droplet size, and its effect was superior to that of ultrasonic time, PHL/WPI ratio and ultrasonic power. Geng et al. also found that the emulsifier concentration was crucial for the formation of emulsion (39). Figures 3D–F demonstrated that high WPI/PHL ratio, long ultrasonic time (>6 min) and suitable ultrasonic power (650 W-750 W) were favorable for the formation of nano-emulsion, which was consistent with the results of section 3.2. The variation ranges of the factors were set within the experimental range, the optimum process conditions were determined based on the model simulation as follows: emulsifier concentration, 1.7%; WPI/PHL ratio, 2.96:1; ultrasonic power, 725 W; ultrasonic time, 7.18 min. In order to adapt to the actual operation, the preparation conditions were adjusted as follows: emulsifier concentration, 1.7%; WPI/PHL ratio, 3:1; ultrasonic power, 700 W; ultrasonic time, 7 min. Under the above conditions, the median diameter of the obtained nano-emulsion was 317.70 ± 5.26 nm, close to the predicted value of 320.20 nm, which confirmed the reliability of the model. The preparation process of PHL-WPI nano-emulsion based on ultrasonic method is simple, time-consuming and practical.

### 3.4. Protective effect of PHL-WPI nano-emulsion on β-carotene

**Figure 4:**
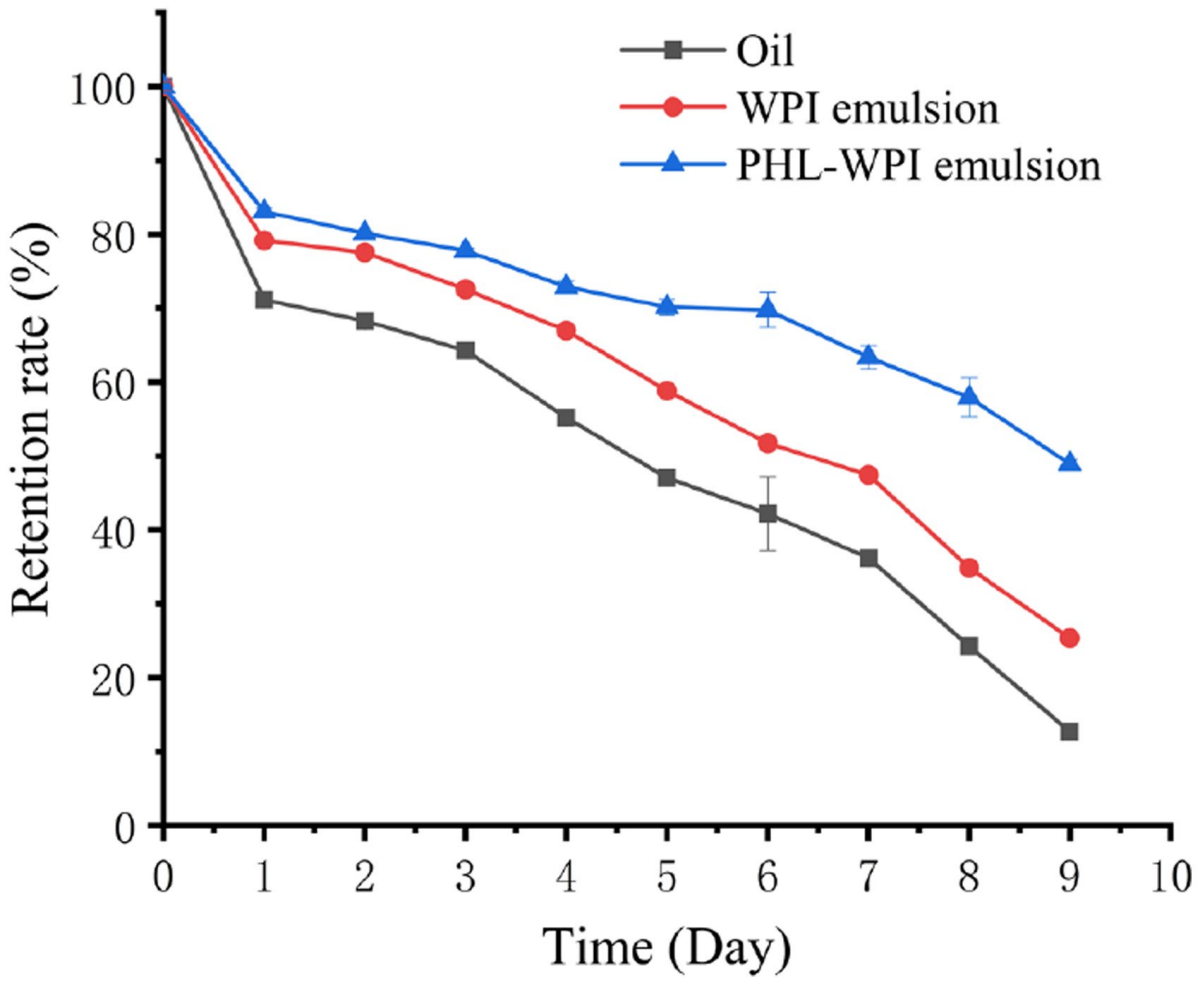
β-Carotene retention rate of oil, WPI emulsion and PHL-WPI emulsion under UV irradiation.

β-Carotene is mainly found in natural fruits and vegetables. It has many physiological functions, such as strong antioxidation, preventing and treating diabetes, reducing the incidence of cardiovascular disease, protecting vision and anti-cancer (40, 41). However, β-carotene has poor water solubility, easy degradation and low bioavailability, which limits its application in food, medicine and other fields. Emulsion can protect β-carotene from the surrounding environment (light, oxygen, temperature and pH), improve the water solubility of β-carotene and enhance its bioavailability. Li et al. fabricated β-carotene-loaded emulsion stabilized by soy protein isolate-arabinoxylan hydrolysate conjugates (42). Falsafi et al. prepared and characterized the β-carotene-loaded emulsions developed by whey protein isolate and gum Arabic for electrospinning (43). In this study, the β-carotene protection capacity of PHL-WPI nano-emulsion against UV irradiation was investigated. As shown in Figure 4, the β-carotene retention rate of corn oil decreased rapidly with UV irradiation, and on the fifth day, its retention rate was 47.02%, while the retention rate of β-carotene in WPI emulsion and PHL-WPI emulsion were 58.84 and 70.14%, respectively. On the ninth day, the retention rate of WPI emulsion was 25.36%, while that of corn oil was less than 13%. Nearly 50% β-carotene was still retained in PHL-WPI emulsion. The protective effect of PHL-WPI emulsion and WPI emulsion was attributed to the certain shielding ability of emulsifier film on the surface of oil droplets to UV radiation. The complexation of WPI and PHL could improve the strength and density of the film, so the protective effect of PHL-WPI emulsion on β-carotene was superior to that of WPI emulsion at the same emulsifier concentration. Li et al. developed an emulsion based on sodium alginate, phloridzin and WPI, and found that the stability and β-carotene protection ability of the emulsion were positively correlated with the content of sodium alginate (12), which was consistent with our results.

## 4. Conclusion

PHL was mainly composed of glucose (47.40 mol%), galactose (19.21 mol%) and arabinose (20.21 mol%) with the weight-average molecular weight of 137.97 ± 4.31 kDa. The mixture of PHL and WPI could induce the formation of nano-emulsion under the action of ultrasound. The optimal preparation conditions were determined by RSM as following: emulsifier concentration, 1.7%; WPI/PHL ratio, 3:1; ultrasonic power, 700 W; ultrasonic time, 7 min. Due to the existence of PHL, the ability of PHL-WPI emulsion to protect β -carotene from UV irradiation was higher than that of WPI emulsion. This study provides a new strategy for the development of honeysuckle leaves.

## Data availability statement

The original contributions presented in the study are included in the article/supplementary material, further inquiries can be directed to the corresponding author.

## Author contributions

YoL: investigation, data curation, methodology, funding acquisition, formal analysis, writing – original draft, and writing – review and editing. BL: investigation, data curation, software, writing – original draft, and writing – review and editing. JY, JR, and YiL: investigation, and data curation. JS: supervision and project administration. XL: writing – review and editing and validation. All authors contributed to the article and approved the submitted version.

## Funding

This work was supported by the Henan Province Key Research and Development Project (no. 221111110300), Scientific and Technological Project in Henan Province (no. 232102310296), and Key Teachers in Henan Province (no. 2019GGJS165).

## Conflict of interest

The authors declare that the research was conducted in the absence of any commercial or financial relationships that could be construed as a potential conflict of interest.

## Publisher’s note

All claims expressed in this article are solely those of the authors and do not necessarily represent those of their affiliated organizations, or those of the publisher, the editors and the reviewers. Any product that may be evaluated in this article, or claim that may be made by its manufacturer, is not guaranteed or endorsed by the publisher.
